# Prevalence, Sociodemographic Characteristics and Risk Factors for Hepatitis C Infection among Pregnant Women in Calabar Municipality, Nigeria

**Published:** 2010-06-01

**Authors:** Clement Ibi Mboto, Iniobong Ebenge Andy, Ogban Ibor Eni, Andrew Paul Jewell

**Affiliations:** 1 Department of Microbiology, Faculty of Science, University of Calabar, Calabar, Nigeria; 2 Department of Pathology, General Hospital of Calabar, Calabar, Nigeria; 3 Faculty of Health and Social Care Sciences, Kingston University and St George’s, University of London, London, UK

**Keywords:** Prevalence, HCV, Pregnancy, Nigeria

## Abstract

**Background and Aims:**

The epidemiology and risk factors for hepatitis C virus (HCV) infection in developing countries where intravenous drug use (IDU) is uncommon its poorly understood. This study therefore aims to determine the prevalence of HCV and its associated risk factors among pregnant women in Calabar municipality.

**Methods:**

A total of 506 out of 716 antenatal care (ANC) patients seen at the General Hospital, Mary Slessor Avenue, Calabar between August and November 2005 and the University of Calabar Teaching Hospital (UCTH) between October and November 2005 were evaluated for their HCV status using the One Step HCV Test kit (Binomial diagnostics, UK), with reference to the subjects’ demographic and behavioural risk factors.

**Results:**

HCV prevalence was determined to be 0.4% (2/506) and was only seen in women aged 38 years and over. Histories of blood transfusion, surgery, involvement in polygamous marriage, sharing of a toothbrush and female circumcision were all non-significant risk factors for the infecion.

**conclusions:**

This study reveals a low HCV prevalence among pregnant women in Calabar municipality with no identifiable risk factor. The study calls for a re-evaluation of the transmission modes of HCV especially in developing countries where intravenous drug use is rare.

## Introduction

Hepatitis C virus (HCV) is a single stranded enveloped positive sense RNA virus belonging to the Flaviviridae family, and is associated with significant morbidity and mortality in men and in women [1]. The infection is a major public health burden worldwide [2]. Evidence of HCV infection has been found in an average of 5% of infants born to HCV mothers and ranges between 5 – 36% of children born to women co-infected with human immunodeficiency virus (HIV) and HCV [3]. Furthermore, maternal HCV carrier status has been reported as an independent risk factor for an adverse perinatal outcome [4].

Despite the clinical sequel associated with HCV, its epidemiology and risk factors are poorly understood, most especially in developing countries where intravenous drug abuse (IDU) is uncommon.

In Nigeria, accurate data on HCV distribution are elusive, and this situation is compounded by a lack of understating of its natural history.In addition, routine diagnosis of infection with the virus is often limited to blood donors. In the Cross River State, little or no published data exist on the distribution of the infection. The practice of blood-transfusion screening of blood for antibodies to HCV (anti-HCV Ab) emerged barely three years ago, and is still limited to only two hospitals located in the state capital. Furthermore, with a very limited number of women participating as blood donors in most parts of the country, including Cross River State, there are no baseline data on the distribution of HCV among women in the state. In this study we sought to determine the prevalence of HCV infection among pregnant women against the backdrop of their sociodemographic characteristics and reported risk factors, as a means of contributing to our understanding of its natural history.

## Materials and Methods

### Study subjects and area

The study subjects were antenatal clinic attendees seen consecutively between August and November 2005 at the General Hospital, Mary Slessor Avenue, Calabar; and at the University of Calabar Teaching Hospital Calabar seen between October and November 2005. The two hospitals serve as the main referral hospitals in Cross River State, Nigeria. The Cross River State shares an extensive border with Cameroon, and has a population of over 3 million. The women were aged 15 to 39 years (mean: 29.3 years).

### Ethical approval

This study has the ethical approval of the ethical committees of both the General Hospital, Calabar and that of the University of Calabar Teaching Hospital, Calabar. In addition, individual consent was a mandatory requirement for participation.

### Administration of the questionnaire and sample collection

Each woman was informed of the study and upon her agreeing to participate, was counselled on a one-to-one basis before a final consent to participate was requested. A questionnaire designed to obtain information on the women’s sociodemographic characteristics, personal and behavioural risk factors was administered to each of those who gave their informed consent to participate. The contents of the questionnaire were explained in detail, to rule out ambiguity or lack of understanding. Explanation was in English or in the local dialect. Very literate women were allowed to complete the questionnaire on their own on the spot, while others had the questionnaire completed on their behalf following an oral interview. Upon the completion of the questionnaire, approximately 5ml of blood were obtained from each woman by venipuncture. Samples drawn were allowed to clot naturally and separated sera were divided into two aliquots, each stored in Eppendorf tubes. Each serum aliquot was kept refrigerated, while the duplicates were frozen. The frozen samples served as replacements for the original, where insufficiency or spillage or contamination of the original refrigerated sample occurred.

### Screening tests for anti–HCV antibodies

Detection of anti-HCV Ab was carried out by using the HCV One Step Strip (Biomill Diagnostic kits, UK). The second aliquot was preserved frozen, and was screened in batches, using the Murex HCV 3.0 enzyme-linked immunosorbent assay (ELISA) kit [5].

## Results

### Study participation

Only 506 out of the total of 716 antenatal care (ANC) women, seen at the two study sites during the study period, consented to participate in the study. Refusal reasons include lack of time, 71.2%; while lack of husband’s consent was 18.4%; other miscellaneous reasons such as fear, nonconformity to personal beliefs, or no reason at all, accounted for the remaining 10.4%.

### Return of the questionnaire

Questionnaire return was close to one hundred percent (99.6%: 506/508). Two women refused to respond to some of the questions asked and were excluded from the study.

### Prevalence of anti-HCV Ab

Overall anti-HCV Ab was only detected in 2 out of the 506 (0.4%) women screened by both the HCV 3.0 One Step Strip and the Murex HCV 3.0 ELISA Test kit. The two positive cases were detected in patients seen at the General Hospital, Calabar (0.5%; 2/398). No anti-HCV Ab was detected among the 208 women seen at the University of Calabar Teaching Hospital, Calabar.

### Sociodemographic characteristics, personal history and HCV distribution among study subjects

A summary of the sociodemographic characteristics and personal history of the women studied is presented in (Table 1).

**Table 1:** HCV prevalence according to age, marital status and number of children

**Parameter**	**No. Involved (n=506)**	**No. HCV Positive (n=2, %)**
**Age (years) **		
≤15	35(6.9)	0
16-23	90(17.8)	0
23-29	192(37.9)	0
20-37	188(37.2)	0
≥38	31(6.1)	2(0.06)
**Marital Status **		
Single	27(5.3)	0
Married	472(93.3)	2 (0.004)
Divorced/widowed	7(1.4)	0
** Trimester of Pregnancy **		
1st	43(8.5)	0
2nd	411(81.2)	2(0.005)
3rd	42(8.3)	0
**Number of Children**		
0-1	62(12.3)	0
2-4	390(77.1)	1(0.3)
≥5	44(8.7)	1(2.3)

### Age, marital status and trimester of pregnancy

Approximately 6.9% (35/506) of the women were 15 years or less, while over half of them (55%, 282/506) were aged 16 – 29 years, 37% (188/506) were aged 30 – 37, and only 6.1% (31/506) were aged 38 years or over. The mean age of the women was 29.33 years. Twenty seven (5.3%) of the women were single parents, while 93.3 % and 1.4% were married or divorced/widowed, respectively. Anti-HCV Ab was detected in only two women, aged 39 and 43, respectively.

### Trimester of pregnancy, number of children and HCV prevalence

Over eighty percent (81.2%, 411/506) of the women screened were in their second trimester of pregnancy and this group accounted for the two positive cases seen. Similarly, more than three quarters of the women had previously had between 2 – 4 children (77.1%, 390/506), while only 8.7 % (44/506) had five children or more and 12.3% (62/506) had either one or no child at all. Anti-HCV Ab was detected in one of the women with four previous children and in another with nine previous pregnancies with seven surviving children.

### Risk factors

Only 5.3% (27/506) of the women reported a history of blood transfusion, while 2.6% (13/506) gave a history of surgery within the past ten years. These groups were not associated with anti-HCV Ab. An HCV prevalence of 0.4% (2/479) and 0.41% (2/493) was detected in those with no history of blood transfusion, and those without any history of surgery within the past ten years, respectively. Similarly, involvement in polygamous marriage, sharing of a toothbrush and a history of tattoos or female circumcision were all not considered risk factors for the distribution of HCV in this study.

Over five percent of the women were single parents 5.37 (27/506) while 93.3% (472/506) were married and 1.4% (7/506) was either divorced or widowed. Anti- HCV Ab was only detected among the married women (0.4%; 2/472) (Table 2).

**Table 2:** Prevalence of HCV according to risk factor

**Parameter**	**No. Involved (n=506)**	**No. HCV Positive (n=2)**
**History of Blood Transfusion within the Past 10 Years**		
Yes	27(5.3)	0(0)
No	479(94.7)	2(0.004)
**History of Surgery within the Past 10 Years**		
Yes	13(2.6)	0(0)
No	493(94.7)	2(0.004)
**Involvement in Polygamous Marriage**		
Yes	50(9.9)	0(0)
No	456(90.1)	2(0.004)
**Sharing of Tooth Brush**		
Yes	24(4.7)	0(0)
No	482(92.5)	2(0.004)
**History of Tattoo**		
Yes	6(0.2)	0(0)
No	500 (99.8)	2(0.004)
**Sharing of Injection Needle**		
Yes	0(0)	
No	506 (100)	2(0.004)
**History of Circumcision**		
Yes	77(15.2)	0
No	429 (84.8)	2(0.005)

### Intravenous drug use/injection needle sharing

None of the women admitted ever being involved in intravenous drug use or in the sharing of injection needles.

## Discussion

Studies of infection with HCV in the West Africa region are becoming important and necessary because of the emerging evidence of high HCV prevalence [6][7] against a backdrop of unidentified risk factors.

The finding of an HCV prevalence of 0.4% (2/506) in this study, though comparatively low, is however similar to the findings of Mboto et al. [8], in a similar study conducted in Gambia. It also similar to the Onakewhor and Okonofua [9] report of an HCV prevalence of 1.86% in 269 booked consecutive antenatal women volunteers attending the University of Benin Teaching Hospital, Benin City. Contrary to these findings, however, Halim and Ajayi [10] in a survey of two hundred and sixty healthy volunteer male blood donors aged 20-54 years, found an HCV prevalence of 12.3% in Benin , the capital city of Edo State, suggesting a major disparity due to a difference in sex. Although the study subjects enlisted by Halim and Ajayi [10] were male blood donors, as compared to pregnant women in this study, both groups belong to the low risk class, most especially in terms of transmission of sexually transmitted diseases (STD).

However, the finding of significantly higher anti-HCV Ab prevalence in males by Halim and Ajayi [10] is similar to the Nwankiti et al. [11], report of an HCV prevalence of 14.36% in apparently healthy individuals with a family history of diabetes in Vom, Plateau State. This sex difference cannot be sufficiently explained against a backdrop of unidentified risk factors, most especially as intravenous drug abuse is rare in this part of the world. However, some studies have shown a significant number of persons clear HCV from their blood after infection, with a higher clearance rate in females than males [12]. We do not know if this higher clearance rate in females can sufficiently explain the low HCV prevalence found in this study and the comparatively higher prevalence reported in men by two independent studies [10][11].

Laurent et al. [13], in a similar study conducted among commercial sex workers and pregnant women in Kinshasa, Democratic Republic of Congo, observed that parenteral transmission predominantly by means of blood transfusion and STI treatment-related injections, may play a major role in HCV distribution, while sex was considered a limited factor. In this study, anti-HCV Ab was not detected in any of the women with a history of blood transfusion. Furthermore, its nondetection in women with a history of surgery within the part ten years, with a history of sharing of a toothbrush, a history of tattoos, and of circumcision, all reveals the insignificant role of these modes of transmission in the distribution of HCV in this region. However, the 0.4% (2/479) of the women who were infected with the virus in this study reported no history of blood transfusion, nor surgery within the past ten years, nor sharing of a toothbrush nor history of tattoos or female circumcision. These persons infected with the virus with no identifiable risk factors sum up the problems associated with HCV studies in the region. several studies have observed significantly high HCV prevalence in subjects with no apparent risk factors (3, 6-8). This finding therefore suggests the need for a longitudinal study that would evaluate some salient cultural practices that may facilitate HCV transmission in the region.

Generally, sexual transmission of HCV is considered to be rare, and some studies have shown that the risk of HCV transmission in heterosexual or monogamous relationships is very rare or even null [14][15][16]. However, HCV transmission is enhanced in persons co-infected with HIV [3][17]. In this study, the spousal HCV or HIV status of these women was not evaluated. There is therefore a need for studies aimed at evaluating couples for their HCV status against a background of their prevailing risk factors.

Age has been argued as a major factor in HCV studies, with infection more predominant in older persons [18][19]. This may be due to the prolonged period of incubation of the virus, or to the fact that more people were infected in the developed world before 1989 [20], when diagnostic facilities emerged. In a study conducted in 2000 in Egypt, Abdel-Aziz [21] found anti-HCV Ab prevalence to increase sharply with increasing age; with persons currently or previously married more likely to be seropositive than those who never married. In this study, anti-HCV Ab was found only in two women, aged 38 and 41 years (mean 38.5years), both married and in their second trimester of pregnancy. The significance of these ages in terms of time of infection and mode of transmission cannot be inferred from the available data and calls for further study.

Some studies have revealed that the detection of anti-HCV Ab may yield a high frequency of false-positive results in people at low risk [22]. The contributory role of this to the HCV prevalence found in this study has no simple explanation.

## Conclusions

This study has provided some baseline data on the distribution of HCV among pregnant women in this part of the country, and reveals a significantly low prevalence, as compared to rates reported in males in some other parts of the country. The infected women were 38 years or over, and have no identifiable risk factors, demonstrating the need for a re-evaluation of the transmission routes of HCV, especially in developing countries, where intravenous drug use is rare.
